# FPGA-Based Weighted DTW Framework with Hybrid Gait Symmetry Index for Real-Time Wearable Gait Classification

**DOI:** 10.3390/s26144644

**Published:** 2026-07-22

**Authors:** Kishore Vennela, Bukya Balaji, Mangali Chinna Chinnaiah, Siew-Kei Lam, Narambhatla Janardhan, Penmetsa Subramanyam Raju, Dodde Hari Krishna, Gaddam Divya Vani, Mudasar Basha

**Affiliations:** 1 Department of Electronics and Communication Engineering, Koneru Lakshmaiah Education Foundation, Vaddeswaram 522302, Andhra Pradesh, India; 183040090@kluniversity.in (K.V.); balaji@kluniversity.in (B.B.); 2 Department of Electronics and Communication Engineering, B. V. Raju Institute of Technology, Medak District, Telangana 502313, India; subramanyamraju.p@bvrit.ac.in (P.S.R.); harikrishna.dodde@bvrit.ac.in (D.H.K.); divyavani.g@bvrit.ac.in (G.D.V.); mudasar.basha@bvrit.ac.in (M.B.); 3 College of Computing and Data Science, Nanyang Technological University, Singapore 639798, Singapore; assklam@ntu.edu.sg; 4 Department of Mechanical Engineering, Chaitanya Bharati Institute of Technology, Gandipet, Hyderabad 500075, Telangana, India; njanardhan_mech@cbit.ac.in

**Keywords:** dynamic time warping, gait classification, FPGA, gait symmetry index, wearable sensors, real-time systems

## Abstract

Gait symmetry analysis has emerged as an important tool in rehabilitation engineering and neurological disorder assessment, as it provides clinically relevant indicators of mobility impairment and gait abnormalities. The proposed framework integrates gait symmetry variability, statistical gait features and Dynamic Time Warping (DTW)-based temporal alignment to enhance robustness against gait variations and irregular walking patterns. A hybrid feature vector comprising DTW similarity scores, the hybrid gait symmetry index (GSI), and statistical gait descriptors was employed to classify gait patterns into five categories: normal, slow, medium, fast, and abnormal. The system was implemented as a wearable edge-computing platform using an NI myRIO device equipped with a tri-axial Inertial Measurement Unit (IMU) mounted on the subject’s body. The onboard FPGA performs real-time signal preprocessing, GSI computation, feature extraction, constrained DTW matching, and gait classification using fixed-point streaming architectures and BRAM-based buffering. Meanwhile, the embedded ARM processor manages TCP/IP communication and transmits real-time gait information to a remote monitoring workstation via a WiFi interface for visualization and analysis. Operating at a clock frequency of 100 MHz, the complete architecture achieves an end-to-end processing latency of approximately 4 ms. The proposed FPGA-based implementation provides low-latency, energy-efficient, and real-time gait analysis, making it well suited for wearable rehabilitation systems, assistive healthcare devices, and continuous mobility monitoring applications.

## 1. Introduction

Wearable sensor-based gait analysis has become an important research area in healthcare monitoring, rehabilitation engineering, elderly assistance systems, and human activity recognition. Recent advancements in Inertial Measurement Units (IMUs), accelerometers, and embedded sensing technologies have enabled continuous and real-time gait monitoring outside laboratory environments. Several studies demonstrated that wearable sensors can effectively estimate gait parameters such as cadence, stride duration, walking speed, balance, and gait variability while maintaining low cost, portability, and energy efficiency [1,2,3,4,5,6,7,8,9]. Compared with conventional motion capture systems, wearable sensing approaches are more suitable for long-term outdoor monitoring and rehabilitation applications.

Recent research has focused on gait-event detection and wearable human activity recognition using hip-mounted, wrist-mounted, and waist-mounted IMUs and smartphones because these positions effectively capture pelvic and center-of-mass motion during walking [10,11,12,13,14,15,16,17,18]. Statistical, temporal, and frequency domain features such as mean acceleration, RMS, cadence, stride duration, energy expenditure and entropy are commonly used for gait characterization and activity classification [19,20,21,22]. Machine learning algorithms including SVM, Decision Tree, Random Forest, and KNN classifiers have shown reliable performance with relatively low computational complexity, making them suitable for embedded healthcare systems [23,24,25].

Deep learning approaches such as CNNs, RNNs, and LSTM networks have further improved wearable gait classification accuracy by automatically extracting discriminative features from raw sensor signals, which involves extensive data collection and processing [26,27,28,29]. These methods provide robust performance under varying walking conditions; however, they require high memory resources, floating-point computation, and increased processing complexity. Such limitations reduce their suitability for low-power wearable FPGA platforms and edge healthcare systems. Under such constrains abnormality estimation gives a new dimension to the challenges [30]. Dynamic Time Warping (DTW) has become a widely adopted technique for gait signal alignment and temporal similarity analysis because walking cycles naturally vary in speed and duration across individuals [31,32]. DTW-based gait comparison methods outperform conventional Euclidean-distance approaches when signals contain temporal shifts or irregular motion patterns. However, conventional DTW algorithms exhibit high computational complexity which limits real-time implementation on lightweight embedded systems.

Gait symmetry analysis is increasingly used in rehabilitation engineering and neurological disorder assessment because they provide clinically meaningful indicators of mobility impairment and gait abnormality [33,34,35]. Reduced gait symmetry and restricted ROM are strongly associated with Parkinson’s disease, stroke rehabilitation, elderly gait instability, and muscular stiffness. Several wearable sensor studies demonstrated that IMU-based systems can effectively identify gait asymmetry and abnormal locomotion patterns in real-time healthcare applications. FPGA-based biomedical signal processing has gained considerable attention due to its parallel processing capability, deterministic timing, low latency, and energy efficiency [36,37,38]. FPGA accelerators have been applied for biomedical feature extraction, lightweight machine learning inference, and real-time wearable healthcare systems. Hardware acceleration is particularly beneficial for computationally intensive tasks such as DTW alignment and feature extraction.

IoT-enabled wearable systems have been developed which utilize wireless pressure sensors to support continuous gait monitoring in rehabilitation settings [39]. Similarly, Ref. [40] proposed an integrated gait monitoring framework that combines a smart foot orthosis, a walking aid, and a mobile application to deliver multimodal feedback during assisted walking. In the similar way, ref. [41] presented a wearable approach for estimating three-dimensional ground reaction forces from insole pressure sensors using probabilistic learning techniques. Although these studies advance wearable gait assessment and enhance rehabilitation capabilities, their methods mainly rely on software-driven processing, which can limit their suitability for low-latency, resource-constrained embedded platforms. Ref. [42] demonstrates that multi-sensor fusion enhances the robustness and reliability of gait assessment across diverse patient populations and environmental conditions. Ref. [43] primarily focuses on the design, fabrication, and characterization of a smart insole without implementing real-time gait-event detection, gait classification, or embedded hardware acceleration. A comprehensive overview of IoT-enabled gait assessment for continuous monitoring in everyday environments is presented in ref. [44]. It discusses system architectures and future opportunities rather than proposing a novel gait analysis algorithm or hardware implementation. Evaluation of the different sensing technologies’ capability to estimate spatiotemporal gait parameters, including gait speed, stride length, cadence, and stride time, is presented in ref. [45] which focuses more on performance comparison and validation.

Existing FPGA gait analysis systems are largely limited to basic step detection and low-complexity accelerometer processing, whereas most DTW- or machine-learning-based gait assessment methods rely on offline or cloud-based computation, limiting real-time wearable deployment. Furthermore, implementing DTW-based gait analytics on FPGA introduces significant challenges including memory-efficient distance computation, streaming feature synchronization, fixed-point arithmetic stability, and low-resource realization of nonlinear operations. The existing literature does not address the integration of BRAM-Based buffering, CORDIC-Based magnitude computation, adaptive gait-cycle segmentation, hybrid gait symmetry estimation, and fully pipelined Q1.15 arithmetic within a single wearable FPGA framework. Therefore, the development of an FPGA architecture for real-time gait symmetry estimation and classification remains a significant research gap for wearable rehabilitation, fall-risk assessment, and embedded healthcare monitoring applications.

The contribution of this work is summarized as follows:

1. A novel weighted DTW-based gait analysis model is defined incorporating adaptive gait-cycle segmentation, hybrid-model gait symmetry analysis, and statistical feature extraction suitable for fixed-point FPGA implementation.
2. A real-time wearable human gait classification system is developed using an NI myRIO platform mounted directly on the subject’s body along with a tri-axial IMU sensor, enabling continuous gait acquisition, embedded FPGA-based gait processing and remote monitoring.
3. A hardware-efficient constrained DTW architecture is implemented using fixed-point arithmetic and pipelined streaming computation to reduce computational complexity latency.

**Figure 1:**
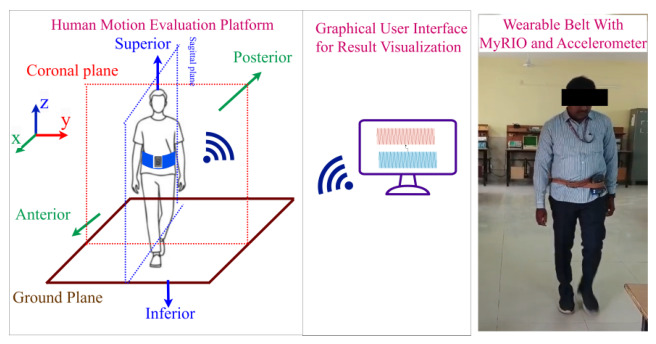
System model.

The proposed work focuses on the hardware implementation of a real-time gait classification framework for wearable sensing applications as shown in Figure 1. The study does not address the design or fabrication of flexible electronic materials, stretchable interconnects, or skin-conformal sensing devices. Instead, the proposed FPGA architecture is intended to interface with commercially available wearable inertial sensors for embedded gait analysis.

## 2. Materials and Methods

### 2.1. Mathematical Model

The framework implements an FPGA-based architecture for real-time gait classification using tri-axial accelerometer signals acquired from a hip-mounted wearable sensor. The complete hardware architecture operates as a sequential streaming pipeline in which each processing stage performs a dedicated operation and synchronously transfers processed data to subsequent modules through valid-signal propagation. The architecture is specifically optimized to achieve deterministic timing behavior, reduced hardware complexity, low-latency execution, and efficient real-time operation suitable for wearable rehabilitation systems, assistive robotics, and embedded healthcare monitoring platforms.

#### 2.1.1. Signal Representation and Preprocessing

The processing flow begins with wearable sensor acquisition, where raw acceleration signals corresponding to sagittal, lateral, and vertical body motion are captured using a tri-axial accelerometer. The discrete-time acceleration vector is represented as

(1) $$a\left[n\right]=[Ac_{x}\left[n\right],Ac_{y}\left[n\right],Ac_{z}\left[n\right]]$$

Since raw inertial signals contain motion artifacts, sensor drift, and high-frequency disturbances, each acceleration axis is independently processed using a low-pass FIR filtering architecture. The filtering operation is expressed as

(2) $${\tilde{Ac}}_{i}\left[n\right]=\sum_{k=0}^{K}h\left[k\right]Ac_{i}\left[n-k\right]$$

where h[k] denotes the FIR filter coefficients and *K* represents the filter order. The filtered acceleration components are subsequently combined using Euclidean magnitude computation to obtain an orientation-independent gait representation:

(3) $$Ac\left[n\right]=\sqrt{{\tilde{Ac}}_{x}^{2}\left[n\right]+{\tilde{Ac}}_{y}^{2}\left[n\right]+{\tilde{Ac}}_{z}^{2}\left[n\right]}$$

In order to ensure FPGA synthesizability and deterministic execution, the square-root operation is implemented using an iterative CORDIC-based architecture, thereby avoiding floating-point hardware overhead.

#### 2.1.2. Streaming Signal Normalization

To reduce inter-subject gait variability and sensor placement inconsistencies, the magnitude signal is normalized using streaming Z-score normalization:

(4) $$Ac_{norm}\left[n\right]=\frac{Ac[n]-\mu }{\sigma }$$

where the running mean (μ) and variance ($\sigma^{2}$) are recursively estimated as

(5) $$\mu =\frac{1}{N}\sum_{n=1}^{N}Ac\left[n\right]$$

(6) $$\sigma^{2}=\frac{1}{N}\sum_{n=1}^{N}{\left(Ac\left[n\right]-\mu \right)}^{2}$$

This normalization stage improves robustness against amplitude fluctuations and enables stable downstream feature extraction.

#### 2.1.3. Adaptive Peak Detection and Gait Segmentation

The normalized gait signal is processed using an adaptive threshold peak detector specifically designed for streaming gait segmentation. Unlike static threshold methods, the adaptive detector continuously updates its threshold according to incoming gait amplitude statistics:

(7) $$\theta \left[n\right]=\theta \left[n-1\right]+\lambda (Ac_{norm}\left[n\right]-\theta \left[n-1\right])$$

where λ controls the threshold adaptation rate.

A gait event is detected whenever the incoming sample satisfies the local maxima condition:

(8) $$Ac\left[n_{k}\right]>Ac\left[n_{k}-1\right]\;\mathrm{and}\;Ac\left[n_{k}\right]>Ac\left[n_{k}+1\right]$$

subject to

(9) $$Ac\left[n_{k}\right]>\theta \left[n\right]$$

In order to suppress false detections caused by oscillatory artifacts, a refractory interval constraint is incorporated:

(10) $$(n_{k}-n_{k-1})>R$$

where *R* denotes the refractory window length. Detected heel-strike events are subsequently used for gait-cycle segmentation:

(11) $$G_{k}=\left\{Ac\left[n_{k}\right],Ac\left[n_{k}+1\right],\ldots ,Ac\left[n_{k+1}\right]\right\}$$

Gait-cycle duration is estimated as

(12) $$T_{k}=\frac{n_{k+1}-n_{k}}{f_{s}}$$

where $f_{s}$ denotes the accelerometer sampling frequency. To support deterministic real-time processing, segmented gait cycles are stored using BRAM-based buffering architectures enabling low-latency memory access during DTW computation.

#### 2.1.4. Weighted DTW Formulation

For temporal gait similarity analysis, the proposed framework incorporates a weighted Dynamic Time Warping (DTW) engine implemented using BRAM-based memory structures. Multiple gait templates corresponding to different walking conditions are stored directly within on-chip memory resources:

(13) $$T=\{T_{1},T_{2},\ldots ,T_{C}\}$$

where *C* denotes the number of gait classes. The local DTW alignment cost incorporates amplitude, slope, and local variance differences:

(14) $$C\left(i,j\right)=w_{a}\left|x_{i}-t_{j}\right|+w_{s}\left|\Delta x_{i}-\Delta t_{j}\right|+w_{v}\left|\sigma_{x,i}-\sigma_{t,j}\right|$$

where $x_{i}$ and $t_{j}$ represent input and template samples respectively, $\Delta x_{i}$ and $\Delta t_{j}$ denote first-order temporal differences, and $\sigma_{x,i}$, $\sigma_{t,j}$ denote local variance estimates. The weighting coefficients satisfy

(15) $$w_{a}+w_{s}+w_{v}=1,\;w_{a},w_{s},w_{v}\geq 0$$

Cumulative DTW distance is recursively computed as

(16) $$D\left(i,j\right)=C\left(i,j\right)+\min\left\{\begin{array}{c} D(i-1,j)\\ D(i,j-1)\\ D(i-1,j-1) \end{array}\right.$$

This DTW cumulative cost matrix D(i,j) has dimensions *N* × *M*, where *N* and *M* represent the number of samples in the input (test) gait sequence and the reference (template) gait sequences respectively. The element D(i,j) stores the minimum accumulated alignment cost computed between the first *i* samples of the input sequence and the first *j* samples of the template sequence. The final DTW similarity metric is obtained as

(17) $$D_{DTW}=D\left(N,M\right)$$

In order to improve throughput, the framework employs multiple DTW engines operating concurrently in parallel hardware pipelines:

(18) $$D=[D_{1},D_{2},\ldots ,D_{C}]$$

where $D_{C}$ denotes the DTW distance associated with gait class *C*.

#### 2.1.5. Hybrid Gait Symmetry Estimation

To evaluate gait symmetry characteristics, the framework incorporates a hybrid gait symmetry estimation module. Amplitude symmetry is initially estimated using alternating (even and odd) gait-cycle peak amplitudes, $P_{even}$ and $P_{odd}$, respectively, and given as

(19) $$GSI_{p}=\frac{|P_{odd}-P_{even}|}{P_{odd}+P_{even}+\epsilon_{p}}$$

Similarly, where $T_{odd}$ and $T_{even}$ represent the durations of two consecutive gait cycles (odd and even cycles, respectively), measured as the time intervals between successive heel-strike events, the temporal symmetry index quantifies the difference in gait-cycle durations, with smaller values indicating more symmetric gait patterns. It is estimated as

(20) $$GSI_{t}=\frac{|T_{odd}-T_{even}|}{T_{odd}+T_{even}+\epsilon_{t}}$$

In the Equations (19) and (20), $\epsilon_{p}$ and $\epsilon_{t}$ are small positive constants added to the denominator to prevent division-by-zero and improve numerical stability during computation. The final hybrid symmetry index is computed as

(21) $$GSI_{h}=\eta GSI_{p}+\left(1-\eta \right)GSI_{t},\;0\leq \eta \leq 1$$

where η controls the contribution of amplitude and temporal symmetry components.

### 2.2. Feature Vector Construction and Classification

In parallel with DTW computation, the framework extracts statistical gait descriptors including mean acceleration, variance, peak magnitude, and gait-cycle duration. These features characterize gait symmetry, motion intensity, and temporal walking behavior. Since individual hardware modules exhibit different processing latencies, a dedicated feature synchronization stage aligns all valid outputs before feature vector generation. The final gait feature vector is represented as:

(22) $$F_{k}=\left[D_{DTW_{k}},\mu_{k},\sigma_{k}^{2},P_{k},T_{k},GSI_{h,k}\right]$$

To improve feature comparability and stabilize fixed-point classifier operation, min–max normalization is independently applied to each feature:

(23) $${\tilde{F}}_{i}=\frac{F_{i}-F_{i}^{\min}}{F_{i}^{\max}-F_{i}^{\min}}$$

This normalized feature vector is subsequently forwarded to a weighted multi-feature classification module. The classifier evaluates similarity between extracted gait features and stored reference templates using a weighted cost function:

(24) $$J_{c}=\sum_{m=1}^{d}\alpha_{m}\left|{\tilde{f}}_{k,m}-{\tilde{f}}_{c,m}^{ref}\right|,\;\sum_{m}\alpha_{m}=1$$

The final gait class is determined using minimum-cost selection:

(25) $${\hat{y}}_{k}=\arg\min_{c\in C}J_{c}$$

The proposed classifier categorizes gait patterns into normal, slow, medium, fast, and abnormal walking conditions. The final decision is encoded as a compact 3-bit output accompanied by a valid synchronization signal for real-time interfacing with wearable healthcare systems, rehabilitation exoskeletons, robotic assistance platforms, and FPGA-based embedded monitoring devices.

## 3. FPGA-Based Real-Time Gait Classification Framework

### 3.1. Hardware Architecture Overview

**Figure 2:**
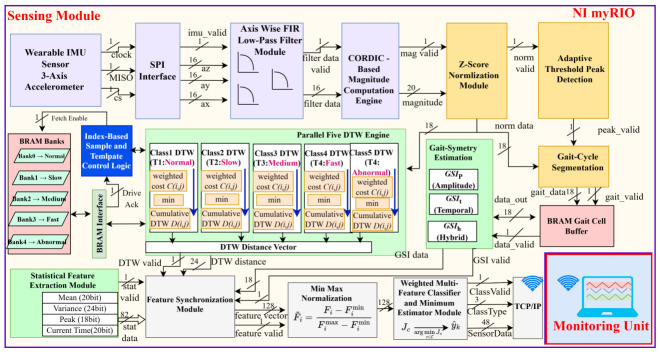
Gait classification system architecture.

The hardware-level process starts with real-time accelereometer data interfaced by myRIO FPGA as shown in the gait classification system shown in Figure 2. This architecture was developed such that the sensor data can continuously flow through successive processing stages. To enable efficient FPGA implementation, all calculations are performed using fixed-point arithmetic. By simplifying hardware realization, this method reduces the required resources.

The parallel FIR filter modules are the first phase, suppressing noise in the collected samples and removing samples due to unnecessary data caused by human motion. The filtered tri-axial data are combined to obtain an acceleration magnitude signal so that they are less sensitive to changes in sensor orientation. The architecture developed for the magnitude normalization stage compensates the variations in signal amplitude caused by differences in walking style and sensor placement.

Using heel-strike events, the collected continuous signal is converted into individual gait cycles. To trace heel-strike events, an adaptive peak-detection mechanism is developed via which individual gait cycles are derived. The derived cycle data is stored in dual-port BRAM buffers, allowing simultaneous data acquisition and feature processing. In order to evaluate the temporal similarity, multiple DTW engine modules are developed that all start processing in parallel. Each engine compares the incoming gait cycle with a predefined reference template in the BRAM. In addition to DTW similarity, gait symmetry and statistical descriptors are also extracted to capture complementary characteristics of walking behavior.

Considering differences in the latency of all the modules run concurrently, a synchronization stage is developed to capture the correct information using a hand-shaking mechanism. The weighted gait classification process is initiated only after proper collection of the data. The weighted classifier module processes the synchronized feature vector, evaluates class-specific matching costs and selects the gait class category with the minimum cost. This implementation provides optimal memory utilization and low-latency gait classification that is suitable for wearable healthcare and rehabilitation systems.

### 3.2. Adaptive Threshold Peak Detector

**Figure 3:**
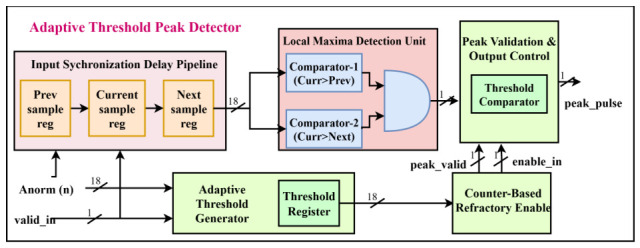
Adaptive peak-detection module micro-architecture.

Figure 3 illustrates the adaptive peak-detection module used for heel-strike identification. Precise detection of events is very important for accurate Gait-Cycle segmentation, particularly under varying walking speeds and signal amplitudes. The detector module combines local maxima analysis with adaptive thresholding and refractory control to increase the robustness during real-time operation. To compute the local maxima, the module stores the neighboring samples using a three-stage delay structure. A heel-strike event is identified whenever the current sample amplitude is more than the neighboring samples. Based on the updated signal characteristics, the module updates its decision threshold rather than binding to a fixed threshold; i.e., the detector continuously using recent signal characteristics. To prevent a false alarm, i.e., multiple detections caused by signal oscillations, a counter-based refractory mechanism is developed. This sub-module activates after each valid event and generates peak_valid and enable_in signals. During this counting interval, additional peak detections are temporarily blocked until the predefined count period is reached. The outputs of the local maxima detector, adaptive threshold unit, and refractory controller are combined to generate the final heel-strike indication peak_pulse, which is subsequently used by the gait-cycle segmentation stage.

### 3.3. Dynamic Time Warping Engine Micro-Architecture

**Figure 4:**
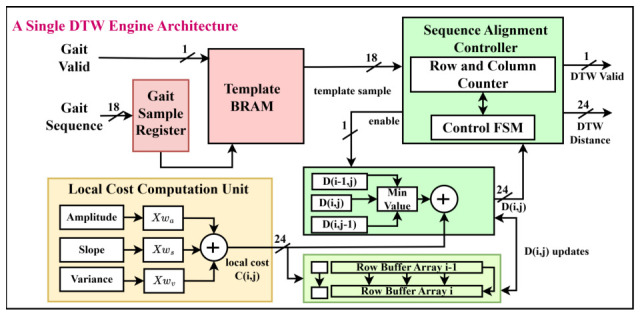
Dynamic time warping engine.

The DTW engine aligns incoming gait cycles with stored reference templates over time; a single DTW engine module is shown in Figure 4. The reference gait templates are stored in dual-port BRAM for parallel access. Using fixed-point arithmetic, the local cost computing unit estimates the similarity cost using difference in amplitude, slope, and variability. Weight values for local cost function are derived from the relative peak amplitudes observed for each gait segment. This allows stronger influence of dominant gait events on the DTW distance estimation.

This subsystem estimates all the accumulated alignment costs associated with vertical, horizontal, and diagonal transitions. The smallest neighboring distance is selected and added to the current cost. An FSM-based controller is used for proper collection and distance estimation among BRAM gait template samples and current gait alongside the local cost estimator.

In each iteration, the DTW distance is updated into row buffers so that it will be available for the subsequent processing. To optimize memory usage, only the current and previous values are stored into row buffers rather than the entirety of the processed data. The final cumulative distance DTWDistance and hand-shaking signal DTWValid are produced when the sequence is complete and further sent to the classifier. Concurrent DTW engines execute matching process with the available gait classes. The number of DTW engines working in parallel is relative to the number of classifications.

### 3.4. Gait Symmetry Estimation

**Figure 5:**
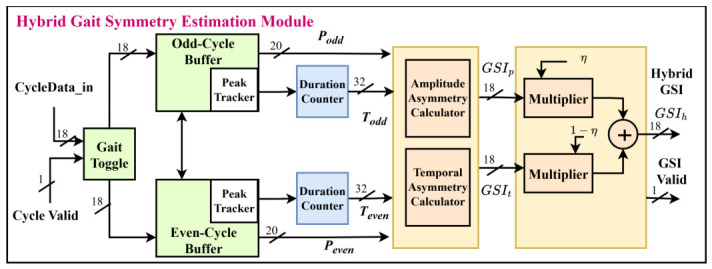
Gait symmetry estimation module micro-architecture.

The subsystem shown in Figure 5 estimates the gait symmetry index, which measures the consistency of alternation between successive gait cycles. For alternating cycles, the system consists of buffers to store consecutive gait cycles, peak tracker units, logic for counter-based peak-to-peak temporal measurement, and circuitry for hybrid fusion. The module estimates the fixed-point-arithmetic-based amplitude and temporal asymmetry, $GSI_{P}$ and $GSI_{t}$ values, respectively, and combines them to measure the HybridGSI value using weighted accumulation η. The estimated value HybridGSI and hand-shaking signal GSIValid are passed on to the feature synchronization stage for further gait class estimation.

### 3.5. Statistical Feature Extraction Micro-Architecture

**Figure 6:**
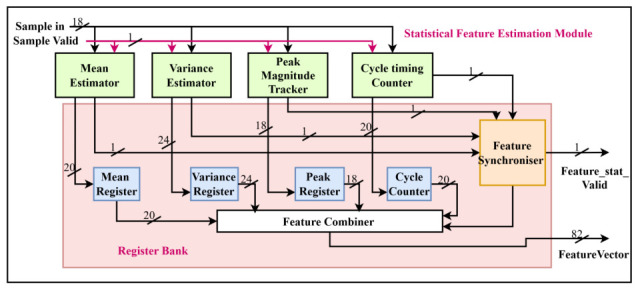
Hardware architecture of the statistical feature extraction module.

Statistical feature extraction is one of the subsystems used in gait classification and is shown in Figure 6. The subsystem is developed to compute statistical features of normalized gait samples parallel to the DTW processing. This architecture is composed of mean and variance estimator units, gait peak tracking logic, and cycle-duration counters.

This subsystem uses the streaming accumulators and fixed-point arithmetic approach to compute the mean and variance of incoming gait samples. The feature synchronizer collects all the computed data from the individual estimators along with individual hand-shaking signals to generate Feature_stat_Valid. The subsystem uses heel-strike event-driven counter implementation for gait-cycle duration measurement and a comparator for peak magnitude measurement. This peak value is the acceleration value at a detected peak. Rather than considering maximum magnitude value of the peak in the register assigned for it, the module computes the mean peak magnitude. To synchronize with DTW and Hybrid GSI estimations, the subsystem generates a hand-shaking signal Feature_stat_Valid along with FeatureVector and provides input to the feature synchronization module.

### 3.6. Weighted Multifeature Classification Architecture

Referencing Equation (22), weighted feature classifiers, the final stage of gait classification, are developed to collected all feature samples for gait classification. Before processing, the synchronized feature vector is normalized to improve the stability of data and balance each feature’s contribution for better classification.

**Figure 7:**
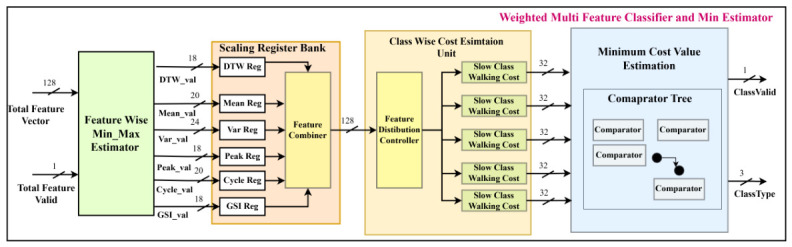
RTL architecture of the proposed weighted Multifeature classifier with minimum value estimator.

The normalized features are distributed simultaneously to the class evaluation branches, each associated with a predefined gait category and template data as shown in Figure 7. In each class, the feature deviations are processed using a weighted mechanism, as defined earlier (Section 2.2), to compute class-specific cost. The minimum value estimator, which is developed as a pipelined comparator tree, computes the smallest matching cost. The minimum-cost class selected as the final gait classification result output ClassType signal along with the hand-shaking signal ClassValid are sent over WiFi for remote monitoring and visualization.

## 4. Results and Discussion

### 4.1. Experimental Protocol

**Figure 8:**
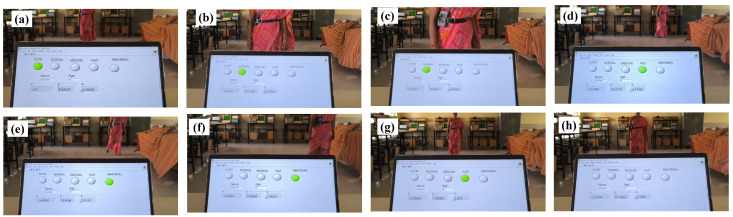
Experimental validation of proposed method for older participants. (**a**–**d**) Identification of gait class based on subject walking pattern. (**e**,**f**) Intentionally altered gait patterns collected from healthy participants to emulate gait abnormalities in older women. (**g**,**h**) Transition trace from fast gait class to sudden stop (no classifier).

**Figure 9:**
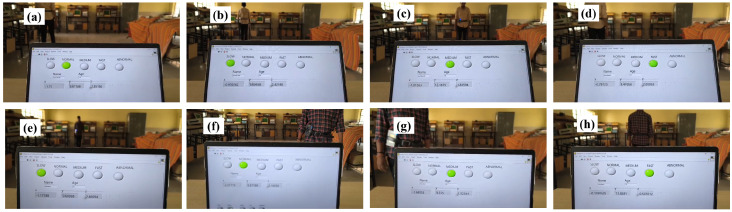
Experimental validation of proposed method in younger people. (**a**–**h**) Identification of gait class based on the young male subject’s walking pattern.

A balanced distribution of subjects was selected to validate the proposed FPGA-based gait classification framework during experimentation. The distribution summarized in Table 1 provides the experimental gait dataset. A total of 60 subjects were part of the study. Among them, 20 subjects were allocated to pilot experiments and 40 subjects were used for final evaluation. Subjects were separated by age group to observe the characteristics of gait patterns and changes and walking behaviors. As shown in Figure 8 and Figure 9, each subject was given instructions to wear a tri-axial accelerometer securely mounted on a hip belt before the experiments. They were instructed to walk continuously for one minute under the specified gait conditions for proper data capturing.

To improve dataset robustness and intra-subject variability, every participant repeated the walking trial three times with a short rest interval. The recorded accelerometer signals from all trials were subsequently used for gait analysis, feature extraction, and classification. A subject-independent evaluation protocol was adopted to assess the generalization capability of the proposed framework. The dataset was partitioned at the subject level such that all gait recordings from a given participant were included exclusively in either the training set or the testing set. This protocol prevents information leakage between training and testing and provides a realistic evaluation of real-world deployment performance.

To evaluate the capability of the proposed classification framework, healthy participants were instructed to deliberately alter their walking behavior to simulate abnormal gait conditions. These intentionally modified gait patterns served as representative abnormal gait samples for algorithm evaluation. No clinically diagnosed pathological gait data were included in this study.

### 4.2. Multi-Class Gait Signal Processing Visualization

**Figure 10:**
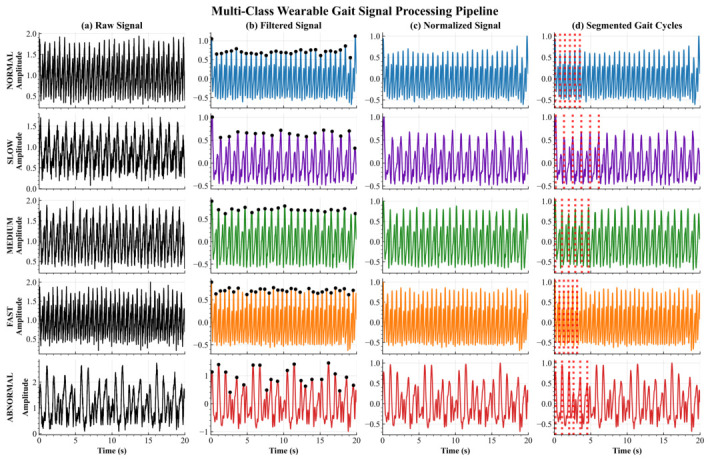
Representative multi-class gait signal processing pipeline showing (**a**) raw acceleration magnitude signal, (**b**) Low pass FIR filtered signal with detected gait events(black dots), (**c**) normalized gait waveforms, and (**d**) segmented gait cycles for normal, slow, medium, fast, and abnormal walking conditions (red dots represent segments).

Experimental results were analyzed with visualization methods and the FPGA resources utilized are given to validate the proposed weighted DTW and gait symmetry approach. Figure 10 showcases observed gait signals for the five categories studied—normal, slow, medium, fast, and abnormal walking—from the experimental data. Each row represents a gait class derived from the execution datasets, while the columns represent the sequential signal processing stages applied in the FPGA-based gait classification framework.

The raw tri-axial accelerometer data is shown lin the first column of the plot in Figure 10. The signals show distinct amplitude and temporal details across gait classes and display greater irregularity in the intentionally altered gait patterns collected from healthy participants to emulate gait abnormalities compared with normal walking. The filter stage eliminates the sensor noise and motion drifts by retaining dominant gait components. These filtered signals are shown in the second column. The gait events detected by the adaptive peak-detection module are indicated by black markers. As mentioned earlier, the signal filtering stage is followed by normalization to improve the consistency of feature extraction and temporal analysis; the results of this are shown in the third column. The vertical dotted red lines shown in the last column in Figure 10 represent cycle boundaries used for subsequent DTW processing. From the figure, it can be seen that normal gaits exhibit relatively uniform cycle spacing, whereas intentionally altered gait patterns show an increased variability in both cycle duration and amplitude measurements.

### 4.3. Baseline Comparison

To quantify the proposed weighted DTW and show the effectiveness of combining the adaptive features, we compared our model against the following baseline methods:

- B1: Threshold-Based Classification: This performs gait classification directly from threshold violations without temporal alignment or feature fusion.
- B2: Conventional DTW: This uses only standard DTW that uses sample-to-sample distance (no GSI, equal weighting for all gait features and local cost estimations).
- B3: Statistical Features Only: This uses only mean, standard deviation, and variance (no temporal sequence information or DTW-based alignment).
- B4: DTW without GSI: This uses a DTW-based gait classification framework with the proposed feature only but without GSI (helps to evaluate the GSI contribution).
- B5: Peak-Based GSI Only: This uses only the GSI evaluation on each peak detection (o feature fusion or DTW ).
- B6: Equal-Weight Feature Fusion: All the gait features are used with equal weight during the distance estimation (showing the importance of adoption of a feature-weighting strategy).
- B7: Support Vector Machine (SVM) Classifier: This provides extracted gait features to a machine leaning-based classifier with a fixed-length feature vector (no temporal sequence alignment).
- Proposed Method: Weighted DTW + Hybrid GSI: This uses weighted feature-level DTW, hybrid GSI and statistical features.

We evaluated (1) accuracy; (2) method precision; (3) recall; (4) F1 score, to check the balance between precision and recall by the classifier; (5) hardware-level entities—latency, power and LUTs occupied; (6) *p*-value. Results are summarized in Table 2. It can be seen that the proposed weighted DTW with hybrid GSI method attained the highest overall classification performance while maintaining moderate latency and power consumption. With high latency and powerful hardware requirements, machine learning methods such as the SVM achieved high accuracy.

### 4.4. Feature Analysis and Resource Utilization

**Figure 11:**
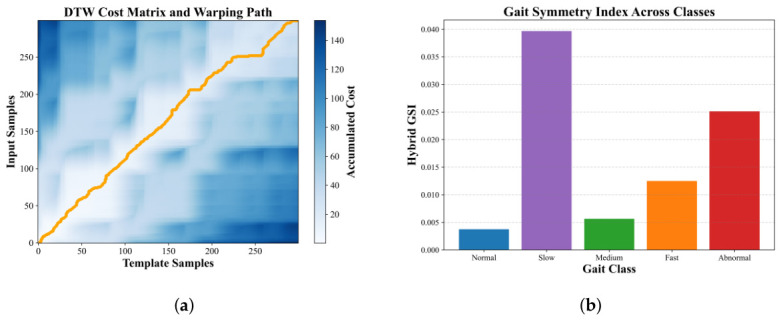
DTW cost matrix and hybrid GSI comparisons. (**a**) Dynamic Time Warping (DTW) cumulative cost matrix and optimal warping path between an input gait sequence and a stored reference template. (**b**) Comparison of the hybrid gait symmetry index (GSI) across different gait classes.

Under conditions such as occurrence of variations in walking speed, the weighted DTW method extracted the gait similarities effectively and improved gait classification robustness compared with conventional DTW. The associated cost matrix is shown in Figure 11a. The wrapping path follows the regions with minimum alignment cost as observed. Hybrid GSI across the gait classes normal, slow, medium, fast, and abnormal are compared in Figure 11b. For normal walking, the symmetry error is lowest, which indicates balanced gait behavior, whereas intentionally altered gait patterns exhibit higher GSI values. This is because of the different amplitude and temporal values in consecutive gait cycles.

The weighting coefficients were determined during an offline optimization stage using only the training dataset. Initially, equal importance is assigned to all feature components. Subsequently, the discriminative capability of each feature is evaluated through cross-validation, and the corresponding weights are adjusted proportionally to maximize classification performance while ensuring that the total weight remains equal to one. After optimization, the final weighting coefficients are stored and employed during real-time inference, thereby eliminating the need for runtime weight adaptation and minimizing computational overhead.

**Figure 12:**
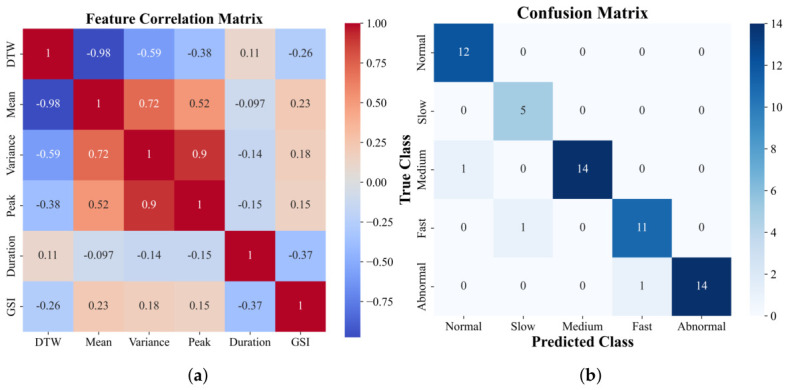
Correlation and confusion matrix observations. (**a**) Correlation matrix of the extracted gait features including DTW distance, mean acceleration, variance, peak magnitude, gait cycle duration, and hybrid gait symmetry index. Color intensity represents the strength of pairwise feature correlation. (**b**) Confusion matrix obtained for the proposed FPGA based gait classification framework under subject independent evaluation. Rows represent true gait classes, while columns indicate predicted classes.

As shown in Figure 12a, the proposed method captures multiple independent features and is capable of discriminating between different walking patterns. From this correlation structure among the extracted gait features, it can be observed that there is a low correlation between other features, but the features correlate well with themselves. Based on the features extracted and applied for gait classification, consistency, i.e., comparison of the actual class with the predicted class, is shown in Figure 12b. Most samples are assigned to their intended gait classes, with a strong diagonal dominance. The observed misclassification between the normal and medium gait reflects the temporal gait characteristics.

The effect of the adaptive weighting in the formation of the weighted DTW local-cost function is shown in Table 3. With uniform weighting it produces lower performance as all the gait descriptors are equally considered in the cost estimation. The proposed adaptive approach focuses primarily on distinctive gait descriptors, specifically temporal slope and amplitude similarity. As a result, a considerable performance increase can be observed across classification accuracy, AUC, and F1-score metrics. A low *p*-value provides statistical validation for these results.

Sensitivity analysis of segmentation window length (*L* samples) and stride length (*S* samples) on classification performance, latency, and BRAM utilization is shown in Table 4. It describes the effect of varying the segment length (*L*) and stride (*S*) on both recognition performance and hardware cost. The overlap value was computed from the segment length (*L*) and stride (*S*) is given as

(26) $$\mathrm{Overlap}\left(\%\right)=\left(1-\frac{S}{L}\right)\times 100$$

From the results, it can be seen that shorter segments provide faster processing and lower memory requirements, but the temporal information captured is not sufficient for the highest classification performance. On increasing the segment length from 64 to 128 samples, both accuracy and F1-score improve across all overlap settings. This indicates that additional gait information contributes to more reliable class separation. The configuration with L = 128 and s = 64 produced the strongest classification metrics while maintaining moderate hardware requirements. Reducing the stride further increases overlap between neighboring segments, whereas removing overlap entirely decreases performance. The increase in segment length further to 256 does not provide improvement compared with 128 and, moreover, increases processing delay and memory usage. Consequently, the performance gains obtained at 128 samples are not sustained for larger segment sizes. To evaluate the effectiveness of the proposed model in recognizing each gait category individually, we evaluated the per-class performance, and results are shown in Table 5. Compared with other gait classes, the abnormal gait class shows high specificity due to amplitude and temporal variations being captured effectively by the hybrid GSI estimator.

Selection of fixed-point arithmetic affects the hardware requirements as well as accuracy aspects in the gait classification process. By balancing the hardware required, power consumed and classification accuracy, 16-bit fixed-point implementation is opted for. As shown in Table 6, lowering the precision reduced hardware overhead but introduced quantization errors. At higher precisions, i.e., 24 bit, hardware requirements are higher, and more power is used. So, the 16-bit implementation provided the optimal trade-off between accuracy, power consumption, and FPGA resource utilization, making it suitable for the gait classification system.

**Figure 13:**
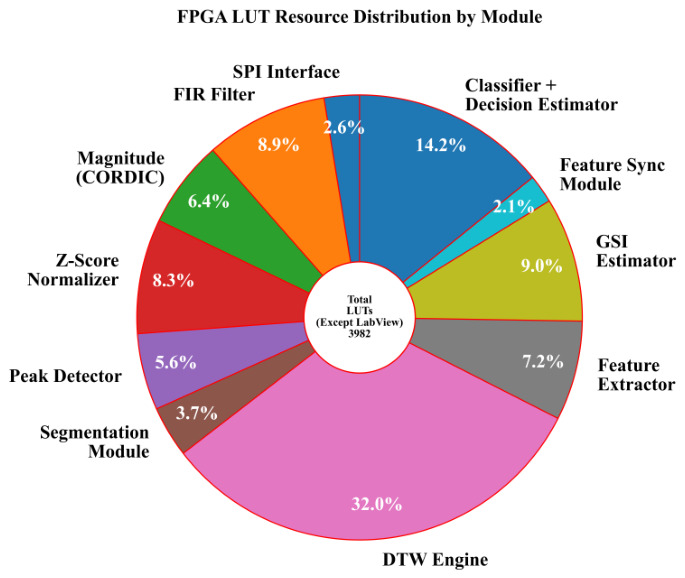
Distribution of FPGA lookup table (LUT) utilization among the major processing modules of the proposed gait classification architecture. The DTW engine occupies the largest fraction of logic resources due to its high number of computations, while the preprocessing and feature extraction modules contribute comparatively lower hardware overhead.

Figure 13 illustrates the contribution of each processing module to the overall FPGA resource utilization. The DTW engine consumes the largest portion of LUT resources because it performs computationally intensive temporal alignment operations. In contrast, signal preprocessing, peak detection, and classification modules require substantially fewer logic resources. These results confirm that the proposed architecture achieves efficient hardware utilization while maintaining real-time gait classification capability.

Table 7 summarizes the FPGA resource utilization of the proposed real-time gait classification architecture implemented on the Xilinx Zynq-7010 platform. The results demonstrate that the complete streaming pipeline achieves efficient hardware utilization while maintaining deterministic real-time processing capability. The FIR filter bank, CORDIC magnitude computation, and Z-score normalization modules introduce moderate DSP and LUT usage because these stages require arithmetic-intensive streaming operations. The adaptive threshold peak detector and gait-cycle segmentation modules consume relatively small resources due to their lightweight control-oriented implementation.

**Figure 14:**
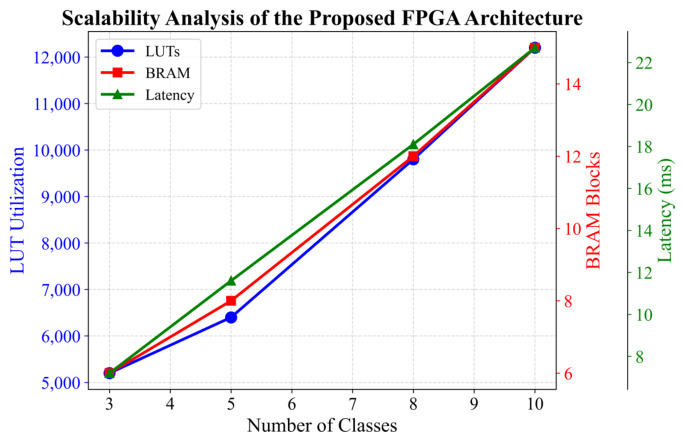
Scalability analysis of the proposed FPGA architecture showing the variation in LUT utilization, BRAM usage, and processing latency as the number of gait classes increases.

As shown in Figure 14, as the number of gait classes increases, the hardware resources required by the proposed architecture also increase. The LUT utilization grows from 5200 to 12,200, and the BRAM requirement increases from 6 to 15 blocks when the number of classes is expanded from three to ten. The processing latency also rises from 7.2 ms to 22.7 ms due to the additional DTW computations required for more gait classes. Even with this increase, the latency remains within the range required for real-time gait analysis, demonstrating that the proposed architecture scales efficiently while maintaining practical hardware resource utilization.

The parallel weighted DTW engines represent the dominant hardware component because recursive operations require more arithmetic and BRAM resources for simultaneous multi-template alignment. However, parallel pipelining significantly improves throughput while maintaining acceptable hardware utilization. The hybrid GSI estimator, statistical feature extractor, and weighted multi-feature classifier introduce moderate resource overhead while enabling accurate gait symmetry analysis and feature-level classification. As the feature fusion synchronization and minimum value decision estimators are implemented using simple control and comparison logic, they only need relatively low resources.

### 4.5. Fixed-Point Precision Analysis

The impact of numerical precision on the proposed gait classification system was evaluated by comparing floating-point and fixed-point implementations. The floating-point model was considered as the reference baseline, providing the highest numerical accuracy with a classification accuracy of 96.3%. When the precision was reduced to 8-bit representation, quantization effects introduced higher deviations in DTW and GSI computations, resulting in errors of 4.7% and 3.9%, respectively. Consequently, the classification accuracy decreased to 91.8%.

Increasing the fixed-point resolution improved the representation of gait features and reduced quantization-induced errors. The 12-bit implementation achieved DTW and GSI errors of 1.5% and 1.2%, respectively, with an accuracy of 94.6%. Further enhancement to 16-bit precision reduced the computational errors to 0.4% for DTW and 0.3% for GSI, achieving an accuracy of 95.8%, which is close to the floating-point performance. These results shown in Table 8 indicate that 16-bit fixed-point arithmetic provides an effective trade-off between computational efficiency and classification performance. Therefore, the 16-bit precision was selected for FPGA implementation, as it minimizes hardware resource utilization while maintaining accuracy comparable to the floating-point reference model.

Table 9 provides a comparison of the proposed gait classification method with related studies. The total architecture occupies only 43.8% of the available DSP resources, 18.3% of BRAM resources, and 36.4% of LUT resources (6400 out of 17,600) on the target Xilinx Zynq-7010 FPGA device, thereby leaving substantial hardware margin for future scalability and additional wearable healthcare functionalities.

## 5. Discussion

The present study evaluates the computational performance of the proposed FPGA framework using commercially available wearable inertial sensors. Mechanical reliability tests, including bending, twisting, repeated wearing, vibration, and impact evaluation, were not within the scope of this work. Future studies will integrate the proposed algorithm with flexible wearable platforms to investigate long-term mechanical reliability under realistic operating conditions.

Table 10 compares the proposed weighted classifier with three commonly used classification methods. The Decision Tree classifier provides the lowest accuracy (92.8%) while requiring a moderate amount of FPGA resources and memory. The Random Forest classifier improves the accuracy to 94.2%, but its implementation requires higher hardware complexity and memory. The Tiny Neural Network achieves an accuracy of 95.1%; however, it also increases the computational and memory requirements.

The proposed weighted classifier achieves the highest classification accuracy of 95.8% with low FPGA complexity, low memory usage, and very low processing latency. This combination makes it well suited for real-time gait classification on resource-constrained FPGA platforms. The results indicate that the proposed method offers improved classification performance while maintaining an efficient hardware implementation.

## 6. Limitations

The proposed gait segmentation and classification framework was evaluated using wearable IMU measurements. Although the obtained results demonstrate promising classification performance, future work will include validation against established gait analysis systems such as optical motion capture, instrumented walkways, and standardized clinical gait assessment scales to further quantify the accuracy of gait-event detection and feature extraction.

## 7. Conclusions

This study presents an FPGA-based weighted DTW and hybrid gait symmetry framework for real-time gait classification using wearable accelerator sensing and NI myRIO FPGA-based processing. The architecture achieves gait classification accuracy by incorporating adaptive preprocessing, weighted DTW cost estimation, gait symmetry analysis, statistical feature analysis and feature fusion with decision making while maintaining efficient hardware utilization. Experimental evaluations with 60 participants of different age groups and genders show the accuracy of the proposed method in gait classification. The baseline comparisons (Section 4.3) quantify advancement in classifying gait patterns effectively. The raw data was collected only from the accelerometer; integrating additional physiological sensing modalities such as electromyography (EMG) would extend the validation for elderly groups with serious gait disorders.

Although the proposed framework demonstrates promising performance for distinguishing normal and intentionally altered gait patterns, further clinical validation involving patients with neurological or musculoskeletal disorders is required before deployment in rehabilitation or clinical decision-support applications. The proposed FPGA-based gait classification framework provides an efficient embedded platform that may support future rehabilitation monitoring, fall-risk assessment, and neurological gait analysis. However, validation on elderly participants and clinical populations remains an important direction for future work.

## Figures and Tables

**Table 1:** Subject demographic distribution used for gait acquisition experiments.

Age Group	Subjects	Pilot	Experimental	Male	Female	Avg. Weight (kg)	Avg. Height (cm)
Below 25	15	5	10	9	6	63.4	168.2
25–40	15	5	10	8	7	67.1	170.8
40–55	15	5	10	7	8	70.6	166.9
Above 55	15	5	10	8	7	72.8	164.5
Total	60	20	40	32	28	68.5	167.6

**Table 2:** Performance comparison of the proposed FPGA-based gait classification framework with baseline methods.

Method	Description	Acc.	Prec.	Rec.	F1	Latency	Power	LUTs	*p*-Value
B1	Threshold-Based Classification	78.4	76.9	75.8	76.3	5.2 ms	1.8 W	2100	0.084
B2	Conventional DTW	84.7	83.5	82.8	83.1	18.6 ms	2.9 W	5400	0.031
B3	Statistical Features Only	81.2	80.6	79.3	79.9	9.8 ms	2.1 W	3400	0.049
B4	DTW without GSI	88.6	87.9	87.2	87.5	21.4 ms	3.2 W	6900	0.018
B5	Peak-Based GSI Only	86.3	85.5	84.7	85.1	14.2 ms	2.6 W	4700	0.026
B6	Equal-Weight Feature Fusion	90.1	89.6	88.9	89.2	17.3 ms	3.0 W	7200	0.021
B7	SVM Classifier	91.4	90.8	90.1	90.4	28.5 ms	4.1 W	8100	0.012
Proposed	Weighted DTW + Hybrid GSI	95.8	95.1	94.7	94.9	11.6 ms	2.4 W	6400	–

Note: Acc. = Accuracy; Prec. = Precision; Rec. = Recall; F1 = F1-score.

**Table 3:** Ablation study on adaptive feature-weight optimization.

Strategy	$w_{a}$	$w_{s}$	$w_{v}$	Accuracy	F1	AUC	Latency	*p*-Value
Uniform Weights	0.333	0.333	0.333	82.1	0.779	0.843	14.1 ms	–
Grid Search (0.05)	0.40	0.35	0.25	87.3	0.848	0.906	12.8 ms	0.021
Proposed Adaptive Weighting	0.45	0.32	0.23	89.8.	0.866	0.927	11.6 ms	0.004

Note: $w_{a}$, acceleration-feature weight; $w_{s}$, stride-feature weight; $w_{v}$, velocity-feature weight; F1, F1-score; AUC, area under the receiver operating characteristic curve.

**Table 4:** Sensitivity analysis over segmentation window length and overlap stride.

*L*	*S*	Overlap	Accuracy	F1-Score	Latency	BRAM
64	16	75%	84.5	0.824	40.9 μs	3
64	32	50%	85.3	0.833	40.9 μs	3
64	64	0%	83.8	0.815	40.9 μs	3
128	32	75%	88.9	0.857	163.8 μs	6
128	64	50%	89.8	0.866	163.8 μs	6
128	128	0%	88.1	0.847	163.8 μs	6
256	64	75%	87.6	0.842	655.4 μs	12
256	128	50%	88.4	0.853	655.4 μs	12
256	256	0%	86.8	0.834	655.4 μs	12

**Table 5:** Per-class classification performance of the proposed framework.

Class	Precision	Recall	F1-Score	Specificity
Normal	96.1	95.4	95.7	97.2
Slow	94.7	95.0	94.8	96.5
Medium	95.2	94.8	95.0	96.9
Fast	96.4	95.7	96.0	97.4
Abnormal	97.1	96.8	96.9	98.1

**Table 6:** Impact of fixed-point precision on classification accuracy and FPGA resource utilization.

Precision	Accuracy	LUTs	DSPs	Power
8-bit	88.2	4200	11	1.9 W
12-bit	93.5	5600	17	2.2 W
16-bit	95.8	6400	21	2.4 W
24-bit	96.0	8900	29	3.1 W

**Table 7:** FPGA resource utilization of the proposed real-time gait classification framework implemented on the Xilinx Zynq-7010 FPGA operating at 100 MHz. Resource percentages are reported relative to the total device capacity.

Module	DSP48E1	BRAM	LUTs	Flip-Flops	Latency
SPI/IMU Interface	0 (0%)	0 (0%)	102 (0.6%)	90 (0.3%)	48 μs
FIR Filter Bank	4 (5.0%)	0 (0%)	354 (2.0%)	402 (1.1%)	360 ns
CORDIC Magnitude Unit	3 (3.8%)	0 (0%)	254 (1.4%)	290 (0.8%)	180 ns
Z-Score Normalizer	2 (2.5%)	0 (0%)	332 (1.9%)	380 (1.1%)	2.56 ms
Adaptive Threshold Peak Detector	1 (1.3%)	0 (0%)	222 (1.3%)	244 (0.7%)	720 ns
Gait-Cycle Segmentation Module	0 (0%)	3 (5.0%)	148 (0.8%)	168 (0.5%)	1.28 ms
Parallel Weighted DTW Engines	10 (12.5%)	4 (6.7%)	1276 (7.3%)	1400 (4.0%)	163.8 μs
Statistical Feature Extractor	2 (2.5%)	0 (0%)	288 (1.6%)	328 (0.9%)	1.04 μs
Hybrid GSI Estimator Module	4 (5.0%)	0 (0%)	360 (2.0%)	400 (1.1%)	910 ns
Feature Synchronization Module	0 (0%)	0 (0%)	82 (0.5%)	102 (0.3%)	180 ns
Weighted Multi-Feature Classifier	3 (3.8%)	1 (1.7%)	500 (2.8%)	558 (1.6%)	1.12 μs
Minimum Value Decision Estimator	0 (0%)	0 (0%)	64 (0.4%)	74 (0.2%)	30 ns
User Logic Pipeline Total	29 (36.3%)	8 (13.3%)	3982 (22.6%)	4436 (12.6%)	4.06 ms
LabVIEW FPGA Framework & Interface	6 (7.5%)	3 (5.0%)	2418 (13.7%)	3132 (8.9%)	–
Complete System Total	35 (43.8%)	11 (18.3%)	6400 (36.4%)	7568 (21.5%)	–
Xilinx Zynq-7010 Capacity	80	60	17,600	35,200	–

**Table 8:** Effect of fixed-point precision on DTW, GSI error, and classification accuracy.

Precision	DTW Error (%)	GSI Error (%)	Accuracy (%)
Floating Point	0.0	0.0	96.3
8-bit	4.7	3.9	91.8
12-bit	1.5	1.2	94.6
16-bit	0.4	0.3	95.8

**Table 9:** Comparison of representative gait analysis and gait classification studies relevant to the proposed FPGA-based DTW gait framework.

Reference	Objective	Sensor Type	Method	Key Contribution	Limitation
[16]	Gait-event detection and parameter estimation	Single waist-worn IMU	CNN and LSTM Seq2Seq models	Single-IMU gait-event and parameter estimation	High computational complexity
[17]	Wearable gait-event detection	Ear-worn IMU	Signal-processing-based event detection	Ear-worn IMU gait-event detection	No classification or symmetry assessment
[18]	Smartphone-based gait-event detection	Smartphone IMU	Self-supervised learning	Label-efficient gait-event detection	Computationally intensive; no hardware acceleration
[22]	Gait pattern identification	Wearable motion sensors	Feature-based classification	Feature-driven gait pattern recognition	Handcrafted features; software-based implementation
[23]	Gait type classification	Smart insole pressure sensors	Deep learning	Multi-class gait classification using smart insoles	Requires specialized hardware and high computation
[25]	Abnormal gait classification	IMU	Machine learning	IMU-based abnormal gait classification	No real-time embedded implementation
[30]	Gait-event detection	IMU	Automatic event-detection algorithm	Accurate heel-strike and toe-off detection	Limited to event detection
[32]	Gait asymmetry analysis	Gyroscope + plantar pressure	DTW-based analysis	DTW-based quantification of gait asymmetry	No classification or hardware implementation
[33]	Gait symmetry evaluation	Clinical gait measurements	Symmetry metrics comparison	Standardized evaluation of gait symmetry	No wearable or real-time implementation
Proposed	Real-time gait classification	Wearable IMU	Weighted DTW + Hybrid GSI + FPGA	Integrates gait-event detection, DTW matching, gait symmetry evaluation, and FPGA acceleration in integrated framework	Evaluation should carried out across people with different pathological gait conditions and FPGA resources required proportional to dataset

**Table 10:** Comparison of the proposed weighted classifier with conventional classification methods for FPGA implementation.

Classifier	FPGA Complexity	Memory	Latency	Accuracy (%)
Decision Tree	Medium	Medium	Low	92.8
Random Forest	High	High	Medium	94.2
Tiny Neural Network	High	High	Medium	95.1
Proposed Weighted Classifier	Low	Low	Very Low	95.8

## Data Availability

Data is available for the experimental validations at https://youtu.be/JiZ8YhsxkOA (accessed on 8 June 2026).
